# Sexual Harassment in Early Adolescence: Findings From a Cross-Sectional Survey in Secondary Schools in England

**DOI:** 10.1177/10778012251391136

**Published:** 2025-10-29

**Authors:** G.J. Melendez-Torres, Rebecca Meiksin, Ruth Ponsford, Nerissa Tilouche, Neisha Sundaram, Joanna Sturgess, Elizabeth Allen, Maria Lohan, Honor Young, Alison Hadley, Rona Campbell, Chris Bonell

**Affiliations:** 1 3286 University of Exeter College of Medicine and Health, Exeter, UK; 2 Department of Public Health, Environments and Societies, 4906 London School of Hygiene and Tropical Medicine, London, UK; 3 Department of Medical Statistics, 4906 London School of Hygiene and Tropical Medicine, London, UK; 4 School of Nursing and Midwifery, 1596 Queen's University Belfast, Belfast, UK; 5 Hitotsubashi Institute for Advanced Study, Hitotsubashi University Kunitachi, Tokyo, Japan; 6 DECIPHer, Cardiff School of Social Sciences, 2112 Cardiff University, Cardiff, UK; 7 Teenage Pregnancy Knowledge Exchange, 5195 University of Bedfordshire, Luton, UK; 8 Population Health Science Institute, 1980 University of Bristol, Bristol, UK

**Keywords:** early adolescence, sexual harassment, sexual-minority, gender, school commitment

## Abstract

There is little research on sexual harassment among younger adolescents or on how rates vary by gender and other student/school characteristics. Drawing on data from 50 English schools, we explored the prevalence and patterning of victimization in the past year among students aged 12–13. Of 7,060 participants, almost a tenth had experienced sexual harassment. Girls, non-binary students, and sexual-minority students reported the highest rates. Student commitment to school was associated with reduced victimization, particularly among straight students and in higher-attaining schools. Sexual harassment is a priority area for intervention, particularly for students facing the highest risk.

## Introduction

While there is no single, widely accepted definition of sexual harassment (Sweeting et al., 2022), this form of abuse can be thought of as unwelcome contact-based or non-contact sexual behavior (Hill & Kearl, 2011; Sweeting et al., 2022), which can take place in person or online (Sweeting et al., 2022). Research findings on the prevalence of sexual harassment vary based on context, such as time, sample, and setting, as well as by methodological factors, such as recall period and the behaviors specified (Sweeting et al., 2022).

While studies suggest that sexual harassment begins early on, there is little empirical evidence on prevalence in early adolescence. Limited data on sexual harassment in UK secondary schools draws on studies of older adolescents aged 16–18 or on broad samples that mainly involve older adolescents. These suggest that physical harassment is experienced by up to three-fifths of girls and up to a quarter of boys, and verbal harassment by up to four-fifths of girls and half of boys (Girlguiding, 2014; Kar et al., 2015; Sweeting et al., 2022; YouGov, 2010). International studies report similar findings, again for older adolescents or broad age-ranges (Attar-Schwartz, 2009; Clear et al., 2014; Hill & Kearl, 2011; Timmerman, 2002; Witkowska & Menckel, 2005).

One study in England reports that 9.5% of students aged 12–14 experience physical and/or verbal sexual harassment often or occasionally, based on a single-item measure, but does not disaggregate by forms of sexual harassment or by participant characteristics (Meiksin et al., 2020). Only a few international and no UK studies have examined sexual harassment among younger adolescents. A representative survey of students aged 11–13 years in New York found that over two-fifths reported verbal sexual harassment victimization (Rolfe & Schroeder, 2020). A Canadian study of students aged 11–14 years found that around a quarter reported verbal harassment (McMaster et al., 2002). A US cohort study of students aged 14 years found rates of sexual harassment victimization of over two-fifths among boys and girls (Chiodo et al., 2009).

International research reports inconsistently on whether girls or boys are most commonly harassed (Attar-Schwartz, 2009; Hill & Kearl, 2011; Rolfe & Schroeder, 2020) and suggests that sexual harassment is more commonly experienced by sexual-minority and ethnically minoritized students (Attar-Schwartz, 2009; Birkett et al., 2009; Clear et al., 2014; Lind et al., 2020). In the UK, there has been little exploration of the associations between participant characteristics and sexual harassment among young people. UK research offers inconsistent evidence about whether girls or boys experience more sexual harassment (Girlguiding, 2014; Sweeting et al., 2022) and little evidence about variation according to other characteristics such as ethnicity and socioeconomic status. One Scottish study found that sexual harassment was more commonly reported by non-heterosexual than heterosexual participants (Sweeting et al., 2022).

Associations between school connectedness and health outcomes among young people are well established. A systematic review of longitudinal research and intervention studies reports that school connectedness is protective against subsequent depression and anxiety and suggests that interventions to strengthen school connectedness can reduce depressive symptoms among adolescents (Raniti et al., 2022). Meta-analytic review evidence suggests that higher levels of school connectedness are associated with less substance use and violence and with lower mental and sexual health risk (Rose et al., 2022). In research on adolescent relationship abuse, school-level school connectedness, caring relationships with staff, opportunities for participation in school, and reporting a sense of school safety were associated with lower levels of victimization (Jain et al., 2018). There is little evidence internationally, however, about how sexual harassment varies with student attitudes to school and school characteristics. Evidence reviewed earlier suggested that sexual harassment can erode student interest in lessons (Hill & Kearl, 2011). Other studies report how sexual harassment varies with school characteristics. A large US study found lower rates of sexual harassment in schools with higher parental education (Crowley et al., 2019), while an Israeli study reported lower rates of harassment in schools with positive school climates (conceptualized as support from teachers, school anti-violence policy, and student involvement in decision-making) and higher parental education (Attar-Schwartz, 2009). US evidence suggests that a positive high school climate can reduce the association between LGBTQ+ student identity and homophobic harassment (Birkett et al., 2009). No previous UK research has explored relationships between either attitudes to school or school characteristics and sexual harassment.

Sociocultural perspectives frame sexual harassment as rooted in power: as a form of abuse that can serve both to establish and perpetuate the power of dominant over more marginalized groups (Burn, 2019). From this perspective, sexual harassment can be conceptualized as an outcome of societal norms that position heterosexual boys and men as dominant over other groups, a conceptualization that is supported by research identifying strong associations between conformity to stereotypically masculine expectations and attitudes and behaviors supporting sexual violence (Burn, 2019; Locke & Mahalik, 2005). Intersectional analyses also suggest that sexual harassment can interact with other forms of oppression to shape experiences of victimization (Crenshaw, 1992). This is the case, for example, where racial discrimination shapes or takes the form of sexual harassment and where racial prejudice undermines support and justice for ethnically minoritized victims (Burn, 2019; Calafell, 2014; Collins, 2000). Sexual harassment can also function to punish deviations from culturally normative forms of gender expressions (Burn, 2019) among sexual- and gender-minority groups as well as among anyone perceived to be in violation of gendered expectations. Among marginalized groups, harms from sexual harassment can add to harms from other forms of harassment and maltreatment to worsen well-being and distress (Burn, 2019).

It is clear that sexual harassment causes significant educational and health harms. US students experiencing sexual harassment report: finding it hard to pay attention in school, talking less in class, changing their seat in class to get farther away from abusers, and not wanting to go to school (Hill & Kearl, 2011; Lipson, 2001). Experiencing sexual harassment in adolescence is also associated with subsequent victimization, while sexual harassment in high school is associated with reduced well-being (Bendixen et al., 2018; Chiodo et al., 2009). In cross-sectional studies, nonphysical peer sexual harassment is associated with higher levels of anxiety, depression, negative body image, and low self-esteem, even when adjusting for experience of sexual coercion and past forced intercourse (Bendixen et al., 2018). Girls tend to report more harm from experiencing sexual harassment than do boys (Bendixen et al., 2018; Hill & Kearl, 2011). For example, higher proportions of girls than boys report not wanting to go to school as a result of sexual harassment (Hill & Kearl, 2011), and moderation analyses find a stronger association between sexual harassment and depressive symptoms among girls than boys (Bendixen et al., 2018).

In 2020, the “Everyone's Invited” initiative collected thousands of online testimonies about rape and sexual abuse (https://www.everyonesinvited.uk/), drawing attention to sexual abuse and harassment in UK schools. This prompted the Office for Standards in Education, Children's Services and Skills (Ofsted) national school inspectorate for England to undertake a review of sexual abuse in schools and colleges, which recommended that schools take whole-school actions to prevent abuse and harassment (Ofsted, 2021). However, we know little about the prevalence of sexual harassment among younger adolescents or how this varies by student and school characteristics, which are the subjects of the present study.

Given the lack of prevalence estimates for sexual harassment from the UK for younger adolescents, our first research question is: what is the prevalence of sexual harassment victimization among students aged 12–13 years in a diverse range of English schools? Given the mixed UK evidence about how sexual harassment victimization varies with gender and the paucity of UK or international evidence about other student and school characteristics, our second question is: how does sexual harassment vary with student gender, sexual orientation, ethnicity, socioeconomic status, and commitment to school, and with school characteristics?

## Methods

This analysis draws on data collected from the baseline (pre-allocation/intervention) survey of a phase-III cluster randomized controlled trial (RCT) of the Positive Choices sexual health intervention among year-8 students (aged 12/13 years) in 50 English mainstream secondary schools (Ponsford et al. 2021b). This research was conducted in line with UK data protection legislation governing data collection and use (*The UK's data protection legislation*) and was approved by the London School of Hygiene & Tropical Medicine ethics committee. Specialist schools for students with special educational needs and disabilities or for excluded students, and schools deemed inadequate by the Ofsted school inspectorate, were excluded. Recruitment involved emailing schools and school networks with follow-up phone calls.

We surveyed students between November 2021 and March 2022 using paper questionnaires. These were completed confidentially, supervised by trained fieldworkers. Teachers were present to maintain order but remained at the front of classrooms, unable to read student responses. We sought informed written opt-in consent from students judged competent by teachers to provide this. We informed parents/carers about the study and enabled them to opt their children out if they wished. We sent students and parents/carers an information sheet prior to data collection. Before surveys, we gave participants who had not already opted or been opted out oral and written descriptions of the study and the opportunity to ask questions. We surveyed absent students by leaving questionnaires and stamped addressed envelopes with schools, liaising with schools to encourage returns.

We measured sexual harassment using a binary measure of whether students had experienced victimization at school in the past 12 months. The question defined this as “unwanted and unwelcome sexual touching or sexual talk, not including behaviors that were liked or wanted such as wanted kissing, touching, or flirting.” A single-item measure was used to limit participant burden among young students. This measure has been piloted in previous studies in similar populations (Meiksin et al., 2020; Ponsford et al., 2021a) and was adapted to specify the timeframe of the past 12 months. We assessed student sexual orientation as straight, gay/lesbian, or bisexual/other (including asexual, unsure/questioning, or other). We assessed student gender as boy (including trans boy), girl (including trans girl), and non-binary. Sensitivity analyses categorized gender as cisgender boy, cisgender girl, and trans/non-binary. We measured these using new measures piloted in previous studies (Meiksin et al., 2020; Ponsford et al., 2021a).

We assessed ethnicity as White, mixed/multiple ethnic groups, Asian/Asian British, Black/African/Caribbean/Black British, Arab, and other, using an established UK Office for National Statistics measure, collapsing this to White versus minority-ethnic given the need for imputation (see below). We assessed family affluence using the Family Affluence Scale (FAS), ranging from 0 to 12, with higher values indicating higher affluence (Currie et al., 2008). We assessed students’ self-reported commitment to school using the Beyond Blue School Climate Questionnaire (Sawyer et al., 2010). Students responded to four statements (“I try hard in school”; “Doing well in school is important to me”; “Continuing or completing my education is important to me”; and “I feel like I am successful in this school”) as: “Yes, totally agree”; “Yes, I agree a bit”; “No, I don’t really agree”; and “No, totally disagree.” This was scored 0–3 as an average across items, with higher values indicating higher commitment. This scale has previously been assessed as having high inter-item reliability and validity in being associated with various health risk behaviors (Shinde et al., 2018).

School-level attainment was assessed based on routine data on public examinations at age 16, divided by 10 to approximate the impact of an average change in each school's attainment of one exam mark. Local deprivation was assessed in terms of schools’ local Income Deprivation Affecting Children Index (Department for Education, 2015).

In our analysis, we first described the outcome overall and by exposure categories for gender, sexual orientation, and ethnicity before turning to model estimation. To facilitate multiple imputation, we collapsed relevant demographic categories into a parsimonious set of binary or ternary variables and used a pragmatic approach with scale scores. Where fewer than a third of items were missing for school commitment, we generated a scale score as the average of non-missing items. Where any FAS items were missing, we coded the overall scale as missing. We subsequently imputed 20 datasets with an unrestricted joint-imputation method in a multilevel context and a variance-covariance model including all analysis variables.

We then conducted analyses to examine how student commitment to school and school characteristics were associated with sexual harassment victimization. Our modeling strategy focused on two-level generalized linear mixed-effects models with students within schools. We first estimated a multipredictor model using all predictors in a random intercept model. We then estimated a series of interaction models crossing school commitment with student characteristics. As a final step, we considered a random slope component for commitment, covaried the random slope with the intercept for the outcome, and regressed intercept and slope on school-level characteristics.

## Results

Recruited schools were in slightly less disadvantaged areas and had slightly lower rates of student poverty than the average for schools in England. Schools were slightly more likely to have good Ofsted inspection ratings. All other characteristics were similar to other English schools **(**Table 1**)**. The survey response rate was 77%. Descriptive characteristics of the sample of 7,060 students across 50 schools are reported in Table 2. The sample was evenly split between those identifying as boys (47.9%) and those identifying as girls (47.9%); the remainder identified as nonbinary (4.2%). Four-fifths (80%) of students reported a straight/heterosexual orientation. The average FAS score was near the middle of the range for this scale. On average, student commitment to school was high (2.46 out of 3).

**Table 1.** Comparison of Recruited Schools With Secondary Schools in England.

		Study trial schools	England average mainstream secondary schools
		Mean (standard deviation (SD) or %)	
Eligible for free school meals (any time during the past 6 years)		23.1 (14.2)	25.8
School-level attainment (data for state schools)		47.5 (8.8)	47.8
School area-level deprivation		0.125 (0.097)	0.148
Ofsted rating	* Outstanding*	14.6 (7)	14.2
	* Good*	66.7 (42)	52.5
	* Requires improvement*	8.3 (4)	12.7
	* Not available*	10.4 (5)	20.6
School sex makeup	* Mixed sex*	88.0 (44)	84.9
	* Girls only*	8.0 (4)	9.3
	* Boys only*	4.0 (2)	5.8

**Table 2.** Sample and Rate of Sexual Harassment Victimization.

Category		% (n)/mean (SD)	Proportion experiencing sexual harassment victimization
Overall estimate of harassment			0.085
School-level attainment		48.7 (9.3)	
School area-level deprivation		0.118 (0.097)	
Gender	*Boys*	47.9 (3,335)	0.061
	*Girls*	47.9 (3,334)	0.096
	*Nonbinary*	4.2 (290)	0.224
Sexuality	*Straight/heterosexual*	80.1 (5,338)	0.064
	*Gay/lesbian*	2.8 (184)	0.199
	*Bisexual/other*	17.1 (1,139)	0.152
Ethnicity	*White*	78.1 (4,880)	0.079
	*Minority-ethnic*	21.9 (1,368)	0.089
Family affluence scale		6.87 (1.64)	
Commitment		2.46 (0.53)	

The overall proportion of students reporting sexual harassment at school in the last year was 8.5% **(**Table 2**)**. The risk of sexual harassment victimization at school was unequally distributed by student-level characteristics. Model results are presented in Table 3. A multi-predictor model (model 1) suggested that girls (OR = 1.52, 95% CI [1.20, 1.91]) and nonbinary students (odds ratio (OR) = 2.32, 95% confidence interval (CI) [1.54, 3.51]) were at higher risk of victimization than boys. Similarly, gay/lesbian, bisexual, or other students were at higher risk of victimization than straight students. Neither ethnicity nor FAS was significantly associated with sexual harassment.

**Table 3.** Correlates of Sexual Harassment Victimization.

Variable			Multipredictor model	Student-level interactions with commitment				School-level interactions with commitment
			Model 1 OR (95% CI)	Model 2 OR (95% CI)	Model 3 OR (95% CI)	Model 4 OR (95% CI)	Model 5 OR (95% CI)	Model 6 OR (95% CI)
**Student-level**	Gender	*Boy/trans boy*	Reference	Reference	Reference	Reference	Reference	Reference
		*Girl/trans girl*	1.52 (1.20, 1.91)	1.36 (0.98, 1.90)	1.51 (1.20, 1.91)	1.52 (1.2, 1.91)	1.52 (1.20, 1.91)	1.53 (1.21, 1.92)
		*Non-binary*	2.32 (1.54, 3.51)	2.71 (1.51, 4.85)	2.38 (1.58, 3.60)	2.33 (1.54, 3.51)	2.33 (1.54, 3.52)	2.36 (1.56, 3.58)
	Sexual orientation	*Straight/heterosexual*	Reference	Reference	Reference	Reference	Reference	Reference
		*Gay/lesbian*	2.99 (1.93, 4.64)	3.01 (1.94, 4.66)	4.17 (2.36, 7.38)	2.99 (1.93, 4.65)	2.99 (1.92, 4.66)	2.92 (1.86, 4.58)
		*Bisexual/other*	2.17 (1.68, 2.80)	2.16 (1.67, 2.79)	2.73 (1.94, 3.85)	2.17 (1.68, 2.8)	2.17 (1.68, 2.80)	2.14 (1.66, 2.78)
	Ethnicity	*White*	Reference	Reference	Reference	Reference	Reference	Reference
		*Minority-ethnic*	1.23 (0.97, 1.55)	1.22 (0.97, 1.55)	1.23 (0.97, 1.55)	1.15 (0.82, 1.59)	1.23 (0.97, 1.55)	1.23 (0.97, 1.56)
	Family affluence scale		1.01 (0.96, 1.07)	1.01 (0.96, 1.07)	1.01 (0.96, 1.07)	1.01 (0.96, 1.07)	1.01 (0.93, 1.10)	1.01 (0.96, 1.07)
	School commitment		0.66 (0.56, 0.78)	0.71 (0.52, 0.96)	0.58 (0.48, 0.71)	0.68 (0.57, 0.81)	0.69 (0.36, 1.32)	0.67 (0.57, 0.77)
	Interaction: commitment and girl			0.85 (0.59, 1.23)				
	Interaction: commitment and nonbinary			1.20 (0.69, 2.09)				
	Interaction: commitment and gay/lesbian				1.61 (0.92, 2.81)			
	Interaction: commitment and bisexual				1.42 (1.03, 1.97)			
	Interaction: commitment and ethnicity					0.9 (0.63, 1.28)		
	Interaction: commitment and affluence						0.99 (0.91, 1.09)	
**School-level**	Attainment		0.75 (0.67, 0.84)	0.75 (0.67, 0.84)	0.75 (0.67, 0.84)	0.75 (0.67, 0.84)	0.75 (0.67, 0.84)	0.67 (0.57, 0.78)
	Deprivation		0.65 (0.18, 2.40)	0.66 (0.18, 2.45)	0.65 (0.17, 2.42)	0.65 (0.18, 2.41)	0.65 (0.18, 2.40)	1.26 (0.27, 5.96)
	Attainment predicts commitment slope							0.85 (0.73, 0.997)
	Deprivation predicts commitment slope							2.86 (0.53, 15.33)
	Intercept-commitment slope covariance							1.05 (0.44, 2.54)

Higher levels of school commitment were associated with lower risk of victimization, with a one-point increase in commitment linked to a 34% decrease in odds of sexual harassment (OR = 0.66, 95% CI [0.56, 0.78]). At the school-level, lower attainment, but not deprivation, was associated with increased victimization; a hypothetical improvement in average school GCSE grade by one mark was linked to a 25% decrease in odds of victimization (OR = 0.75, 95% CI [0.67, 0.84]). Sensitivity univariate analyses categorizing gender as cisgender boy, cisgender girl, and trans/nonbinary did not change this pattern of associations.

Interaction models using student-level variables did not suggest that the significant association between school commitment and harassment was moderated by gender (model 2), ethnicity (model 4), or affluence (model 5), but did suggest that this association was significantly moderated by sexual orientation (model 3). Specifically, significant interaction terms for school commitment and sexual orientation (specifically for bisexual students) suggested that the association between higher school commitment and lower likelihood of reporting sexual harassment was attenuated for sexual-minority students, with a calculated OR of school commitment for gay or lesbian students of 0.93 and for bisexual students of 0.82. It is notable as well that the association between school commitment and sexual harassment decreased in magnitude from 0.66 across the population to 0.58 for straight students when effect modification was considered. Standard errors for school commitment in the model considering the interaction between commitment and FAS (model 5) were considerably larger, suggesting important collinearity between school commitment and FAS.

Finally, a random slope model examined interactions between commitment and school-level variables (model 6). The regression of within-school slope for commitment on school-level attainment was significant, suggesting that each increase in school-level attainment of one mark decreased the association between school commitment and sexual harassment by 15% (OR = 0.85, 95% CI [0.73, 0.997]). In other words, in higher-performing schools, students reporting higher levels of commitment were less likely than similar students in lower-performing schools to report sexual harassment victimization at school in the last 12 months.

## Discussion

### Main Findings of This Study

A few international but no UK studies have examined sexual harassment among younger adolescents, finding rates slightly lower than among older adolescents (Chiodo et al., 2009; McMaster et al., 2002; Rolfe & Schroeder, 2020). UK evidence is also unclear whether girls or boys experience more harassment (Girlguiding, 2014; Sweeting et al., 2022). There is only one study of how harassment varies with sexual orientation, suggesting non-heterosexual students experience more victimization (Sweeting et al., 2022). There is little evidence about how sexual harassment varies with student attitudes to school and with school characteristics, with plausible mechanisms involving harassment eroding student commitment to school (Hill & Kearl, 2011) and with positive school climates being associated with lower risk of harassment (Attar-Schwartz, 2009). We found that sexual harassment victimization was quite common among younger adolescents, particularly among students less committed to school, girls, and sexual-minority students.

Almost a tenth of students aged 12–13 years had already experienced sexual harassment at school in the previous year. This is lower than reported in UK studies involving older adolescents (Girlguiding, 2014; Meiksin et al., 2020; Sweeting et al., 2022; YouGov, 2010) but still of concern given the adverse consequences arising from victimization, which may be more profound for exposure earlier in life (Kar et al., 2015). The reported prevalence may also underestimate the ongoing prevalence of sexual harassment among this age group, given that participants spent less time in school during the reporting period as a result of the COVID-19 pandemic. It is also possible that COVID-19 prevention actions in re-opened schools, such as one-way systems and “bubbles,” may have reduced the potential for sexual harassment.

In contrast to some other UK studies (Barter et al., 2017; Sweeting et al., 2022), we found that girls (and in the present study, also nonbinary students) were at higher risk of victimization than boys. In line with other UK research (Sweeting et al., 2022), we found that sexual-minority students were at higher risk of sexual harassment victimization than straight students. Although some research suggests that ethnically minoritized girls and young women are at higher risk of sexual harassment (PLAN International, 2021), we found no associations between ethnicity or FAS and sexual harassment in school.

Our study for the first time examines cross-level interactions between school and student characteristics in their association with sexual harassment. Higher school-level attainment was associated with lower risk of harassment. However, there was no association between school-level deprivation and harassment victimization. These findings might suggest that school-level associations reflect specific, potentially modifiable, processes concerning how successfully schools engage students, rather than merely the socio-demographic profile of school intakes.

In our analysis, higher student commitment to school was associated with lower risk of victimization. This association was moderated by sexual orientation such that the association between school commitment and victimization was attenuated among gay/lesbian and bisexual/other-identified students. The association of student commitment to school with reduced victimization was also moderated by school-level attainment, such that the relationship was attenuated in lower-attaining schools.

It may be that sexual-minority young people are particularly vulnerable to sexual harassment that functions to “police” what are seen as (in)appropriate forms of gender expression (Burn, 2019), such that any relationship between higher school commitment and lower likelihood of sexual harassment is overwhelmed by the adverse impacts of heteronormativity and homophobia on sexual-minority students (Cosma et al., 2023).

### Implications for Research and Policy

The most important policy implication of this study is that schools should take action to prevent sexual harassment, and that such work should include early adolescents. A recent systematic review of school interventions to prevent gender-based violence, including sexual harassment, suggested it is harder to prevent gender-based violence outside dating relationships, probably because such behaviors are normative in schools. There was some evidence that the most promising approach involves whole-school interventions aiming to transform school culture and increase student belonging (Farmer et al., 2023). These findings align with sociocultural perspectives conceptualizing sexual harassment as rooted in, and perpetuating, entrenched hierarchies of power (Burn, 2019). The call for whole-school approaches resonates with our finding that school commitment is associated with lower risk of sexual harassment. However, longitudinal research is needed to explore temporality in the relationship between school commitment and sexual harassment reported in this cross-sectional study.

Our findings also suggest that if improving student commitment might be protective against sexual harassment, it may be less so among sexual-minority than straight students. This suggests that school interventions must also actively challenge homo- and trans-phobic norms using evidence-based approaches (Marx & Kettrey, 2016; Schlief et al., 2023). Our findings suggest that interventions are likely to be particularly important in lower-attaining schools with higher rates of victimization. Given the limited evidence on effectiveness, research is needed on which interventions are most effective in preventing sexual harassment overall and by student subgroups. The current UK policy interest in strengthening the role of schools in addressing sexual harassment should encourage school leaders to develop preventive work and researchers to seek funding to trial new approaches.

### Limitations of This Study

Participating schools were not recruited through a random probability sample. The schools were rated by government inspectors as performing slightly better on average than other English schools and were in areas of slightly less socioeconomic disadvantage, but were similar on other characteristics. To reduce participant burden, we used a single-item measure of sexual harassment, which did not differentiate verbal and physical harassment and did not explicitly refer to online forms of sexual harassment. It may have been less sensitive as a result (Sweeting et al., 2022). However, our measure did use a clear definition of sexual harassment, was based on a measure piloted in previous studies with a similar population (Meiksin et al., 2020; Ponsford et al., 2021a), and identified the experience of sexual harassment in nearly a tenth of participant students. A more sensitive, multi-item measure might have identified a still higher prevalence among our sample. Ethnic categories were collapsed to White and minority-ethnic categories to ensure sufficient sample sizes for subgroup analyses, which prevented a more nuanced analysis of sexual harassment victimization by ethnicity.

Our sample of transgender students was not large enough to analyze separately, and we included these students with cisgender students of the same self-reported gender. However, sensitivity univariate analyses categorizing gender as cisgender boy, cisgender girl, and trans/nonbinary did not change the pattern of associations found in our primary analyses. Our analyses rely on cross-sectional data and cannot test hypotheses about causality.

## Conclusion

Nearly one tenth of UK students report experiencing sexual harassment at school in the past year by age 13, with girls and nonbinary and sexual-minority young people at disproportionate risk. Schools should take action to prevent and mitigate the effects of sexual harassment from an early age. Future research should develop and test the acceptability and effectiveness of whole-school interventions to this end, ensuring that these address sexist, homophobic, and transphobic norms underpinning perpetration. Evaluations should assess impact by subgroup and explore the temporal relationship between school commitment and the effects of school-based sexual harassment interventions.

## Abbreviations

CI: confidence interval
FAS: Family Affluence Scale
Ofsted: Office for Standards in Education, Children's Services and Skills
OR: odds ratio
RCT: randomized controlled trial
SD: standard deviation
